# Selective isolation and genomic characterization of biopolymer producer—a novel feature of halophile *Brachybacterium paraconglomeratum* MTCC 13074

**DOI:** 10.1186/s43141-023-00484-y

**Published:** 2023-02-28

**Authors:** Teja Mandragutti, G. Sudhakar

**Affiliations:** 1 Department of Biotechnology, Andhra University, Visakhapatnam, 530003 India; 2 Department of Human Genetics, Andhra University, Visakhapatnam, India

**Keywords:** Biopolymers, Molecular characterization, GC-MS, FAME analysis, *Brachybacterium paraconglomeratum*

## Abstract

**Background:**

Biopolymers like polyhydroxyalkanoates (PHA) are the best natural macromolecules to use as alternative to the synthetic polymers. Many prokaryotes accumulate PHA as cytoplasmic intracellular granules and their accumulation is triggered by starving conditions. The PHAs are ecofriendly and used to create biodegradable plastics. The microbial synthesized PHA had acquired global importance in industrial and biomedical sectors.

**Results:**

Ten different bacterial strains were isolated for the screening of PHA producers from the estuarine region of the Bay of Bengal, Suryalanka in Bapatla. A yellowish slimy circular colony known as M4 is actively growing on selective minimal media and was screened for polymeric granules in its cytoplasm using Sudan Black B and confirmed with the fluorescent dye Nile blue A. All of the isolates were biochemically tested and isolate M4 is the most capable of growing at high NaCl concentrations (3.2 percent) and tests positive for catalase, methyl red. The M4 strain revealed clear hydrolysis of gelatin, starch, and casein. The 16S rRNA sequencing revealed that M4 is 99.72% of identity to *Brachybacterium paraconglomeratum* LMG 19861(T) in BLAST and the obtained strain was assigned with accession no. MTCC 13074 and deposited in NCBI with accession no. MW899045. The chief cellular fatty acids found in M4 were C14:0, C15:0, C16:0, C18:1cis-9, C18:0, iso-C15: 0, iso-C14: 0, anteiso-C17: 0 and C18:1-7. Crotonic acid formation from M4-PHB extract was detected at 235nm in a UV spectrophotometer. Methanolysis was done, and derivatives of polyhydroxybutyric acid (PHB) in the extract were analyzed using GC-MS. Increasing viscosity was seen in the extracts which confirms the presence of polymer in the extracts. Thermogravimetric analysis was studied to determine the thermal profile of the PHB in the extract of M4.

**Conclusion:**

In the study, the selective screening and extraction of ecofriendly PHB from M4 strain was highlighted. *Brachybacterium paraconglomeratum* is a novel strain showed its uniqueness by producing few monomeric derivatives of PHB. The strain was reporting for the first time as PHA producer. *B. paraconglomeratum* has promising characteristics according to its metabolic profile. In addition, this study also helps to understand the diversity of bacteria isolated from marine sources.

## Background

Polyhydroxyalkanoates (PHA) are macromolecules produced from microbiota by consuming different carbon sources. These are ecofriendly and can be used to make biodegradable plastics. Degradable polymeric biomaterials are preferred because of their biological, biomechanical, and physiochemical properties. For a few decades, petroleum-based plastics have been produced and its resistance to biodegradation has created a severe environmental challenge for solid waste management [1]. Biotechnological research is increasingly focusing on the creation of biodegradable polymers as an alternative to petrochemical plastics which are still in wide usage [2].

Over the past few decades, researchers have been working on developing biodegradable polymers using various marine microbial species because of their potential to reduce fermentation and recovery costs of biopolymer. Microbial polymers have the potential to mold into an application, there are already many intriguing applications in the medical and other domains [3, 4]. Microbial bioplastics made up of polylactic acid (PLA) and polyhydroxyalkanoates (PHA) are completely degradable and provide a variety of unique material properties and commercial opportunities [5].

PHAs are the most promising biopolymers because they are nontoxic, biodegradable, and biocompatible and have qualities similar to traditional plastics [6]. PHAs are the polyesters of various hydroxyalkanoates synthesized by microorganisms such as marine actinomycetes, algae, and halophile bacteria. They are strong candidates as biodegradable polymers because they are completely degraded into water and carbon dioxide by microorganisms when they are discarded into nature [7]. Polymer ranges in number of carbons (4 to 14 atoms) and the sort of monomeric units determines structurally whether they are homopolymers or heteropolymers [8]. In circumstances like nutritional imbalance and in stress, the bacterial cells synthesize PHA as stored energy granules in its cytoplasm as a part of defence mechanism [9]. PHAs are implicated in medical, pharmacy, agriculture, food processing, and paint industries [10]. Poly-3-hydroxybutyrate (P3HB or PHB), the most well-known type of PHA, accumulates in many microbes by binding hydroxybutyrate monomers with ester bonds. They are completely linear isotactic structure with 60–70% crystalline content [11]. The analytical studies on physiochemical properties reveal the nature and structural composition of the PHA derivatives.

Marine bacterial species such as *Vibrio harveyi* [12], *Micrococcus* species [13], *Halomonas* elongate [14], and *Bacillus megaterium* [15] have been found to be potent producers of PHB. *Brachybacterium paraconglomeratum* is a species of Gram positive, non-motile, facultative anaerobe, yellowish brown pigmented bacterium which belongs to the phylum Actinobacteria. The cells appear coccoid during the stationary phase and irregular rods during the exponential phase. Although *B. conglomeratum* and *B. paraconglomeratum* are similar in their physiological characteristics, they can be differentiated on the basis of their cellular fatty acid patterns and low levels of DNA-DNA hybridization. To date, only a few reports on the commercialization of PHAs produced by marine microbes and halophiles are available. As a result, the current study was undertaken to determine the strain’s novelty in producing biopolymer.

## Methods

### Isolation and screening of PHA-producing bacteria

Bacterial strains are isolated from the mangrove region of Bay of Bengal, Suryalanka, India. The collected soil samples were transferred to laboratory and stored at −20°C until further use. The soil samples were subjected for serial dilution; initial isolation was done on nutrient agar medium with dextrose. PHA producers were further screened on minimal media, the composition includes dextrose 10.0g/L, ammonium sulfate 1.0g/L, dipotassium phosphate 7.0g/L, monopotassium phosphate 2.0g/L, sodium citrate 0.5g/L, magnesium sulfate 0.1g/L, and final pH (at 25°C) 7.0±0.2 and incubated at 37°C.

### Presumptive test

Isolation and detection for PHA producers using lipophilic stain Sudan black B is a presumptive test [16]. Stain was prepared by dissolution of 0.3 g of dye in 100 ml of 70% ethanol. Stain was flooded on the colonies in selective medium; after 30min, the excess dye was decanted and washed with 100% ethanol, and colonies appear bluish black and accredited as PHA-positive strains.

### Confirmative test

A selective stain, Nile blue A, fluorescent dye is used for the detection of PHB granules [17]. Dye at 0.5μg/ml of selective media was composed and sterilized. The initially screened isolate M4 was streaked on the plates and incubated at 35°C for about 96h. As a positive mark, the colonies emit orange yellow fluorescence under a UV transilluminator.

### Biochemical characterization

The series of biochemical tests were done for all the isolates according to Bergy’s manual, and the activities of M4 were tabulated.

### Genomic profiling of bacteria

The Zymo research (ZR) fungal/bacterial DNA Mini Prep kit was used to isolate genomic DNA from the M4 pure culture. The 16S rRNA gene was amplified by PCR using universal primers 27F AGAGTTTGATCCTGGCTCAG, 357F CTCCTACGGGAGGCAGCA and 786R GATTAGATACCCTGGTAG, 1492R TACGGYTACCTTGTTACGACTT respectively. The PCR conditions for amplification of the 16S rRNA gene were as follows: 5 min of initial denaturation at 94°C, followed by 35 cycles of 1min denaturation at 94°C, 1 min annealing at 56°C, 2min extension at 72°C, and 10 min final extension at 72 C. On a 1% agarose gel, the PCR product was visualized. QIA quick Gel Extraction Kit was used to gel elute and purify the PCR amplicon. The Sanger DNA sequencing method was used to sequence the purified PCR product [14]. Finch TV software version 1.4 was used to visualize and analyze the obtained sequences. The nucleotide sequences of the 16S rRNA gene were compared using the BLAST tool in NCBI and EzBiocloud (http://www.ezbiocloud.net). 

### Fatty acid methyl ester analysis (FAME) of M4

The extraction and analysis of the fatty acid methyl ester profile of M4 was done using GCMSQP2010, SHIMADZU. First, the isolate was grown on Nutrient Agar for 24 h at 35 °C. Forty milligrams of culture was transferred to a Teflon-lined, screw-caped tube and mixed with 1 ml of saponification solution containing 3.75 M NaOH in MeOH:H20 to extract whole lipids. The test tube was vortexed for 5 min and placed in a water bath at 100°C for 25min. Following the incubation period, the test tubes were cooled immediately. Then, 2 ml of methylation solution containing 6.00 M HCl: MeOH (1: 0.85) was added, vortexed, and incubated for 10 min at 80 °C in a water bath before being immediately rinsed with cool tap water. Following that, 1.25 ml of the extraction solution (1:1) hexane: methyl-tert butyl ether was added to the extract. Three milliliters of the washing solution containing 0.3 M NaOH was added and gently mixed for 5 min to remove acidic residual reagents. Finally, the organic phase was transferred from the glass tube to a vial for automated sample injection with the carrier gas alone. Internal standard was prepared to a concentration of 1mg/ml from 10mg of heneicosanoic acid methyl ester (C21:0) [18]. For about 3 h, the column runs at high temperature. The obtained data was searched in the NIST107.LIB Library.

### Extraction of PHA

The isolate M4 was cultured in dextrose-rich and nitrogen-deficit minimal media for about 96h at 35°C at constant 150rpm. Now 5ml culture was taken and centrifuged at 10,000rpm for 10min. The supernatant was discarded the pellet was treated with 2.5ml of 4% sodium hypochlorite for digestion and 2.5ml of hot chloroform, incubated at 37°C/H. Three different phases were observed the upper phase contains sodium hypochlorite was discarded, middle phase contain chloroform with cell debris, and the bottom phase PHA with chloroform was collected, further followed by extraction with hot chloroform and precipitated with 1:1 ethanol and acetone. The precipitate was evaporated to attain PHA crystals or powder [16].

### Crotonic acid assay

This was done according to the procedure described by Law and Slepecky. The extracts of PHA were treated with con.H_2_SO_4_ and heated at 100°C for 10min; as a result crotonic acid was formed. The concentration of crotonic acid was observed at 200 to 600 nm in a UV spectrophotometer [16].

### GC-MS confirmation of PHA

The final extract is prepared for GC-MS. Methanolysis method described by Juengert et al. [11] was chosen for the GC–MS analysis. In a screw-capped glass bottle (20 mL capacity) with a polytetrafluoroethylene cap, 10 mg of extracted PHB, 1 mL chloroform, and 1 mL acidified methanol (15% methanol in H_2_SO_4_) are mixed, heated in a water bath at 100 °C for 2 h. After incubation, the bottle was filled with 1 mL chloroform containing an internal standard 0.2% methyl benzoate v/v and 1 mL deionized water for phase separation. The bottom organic phase was collected and dehydrated with anhydrous Na_2_SO_4_, and an aliquot of 1μl was injected into the Shimadzu GC–MS QP2010S gas chromatograph, which was equipped with a Rxi-5Sil MS (30 m × 0.25 mm × 0.25 μm).

### Physical parameters

#### Thermogravimetric analysis (TGA)

The analysis was carried out with an indium-calibrated TGA apparatus (Mettler-Toledo, TGA/SDTA 851, Columbus, OH, USA). About 5 mg of dried extract was used in the study. The analysis was conducted by increasing temperature from 30 to 400°C at 10°C/min under nitrogen flow [19].

#### Viscosity

The viscosity was measured for culture broth as well as for the extracts of M4 according to the Lovis method using a rolling ball viscometer (Antan paar, Germany). The capillary tube was filled with the sample and allowed to measure the viscosity at 37°C.

## Results

### Isolation and screening of PHA producers

**Fig. 1:**
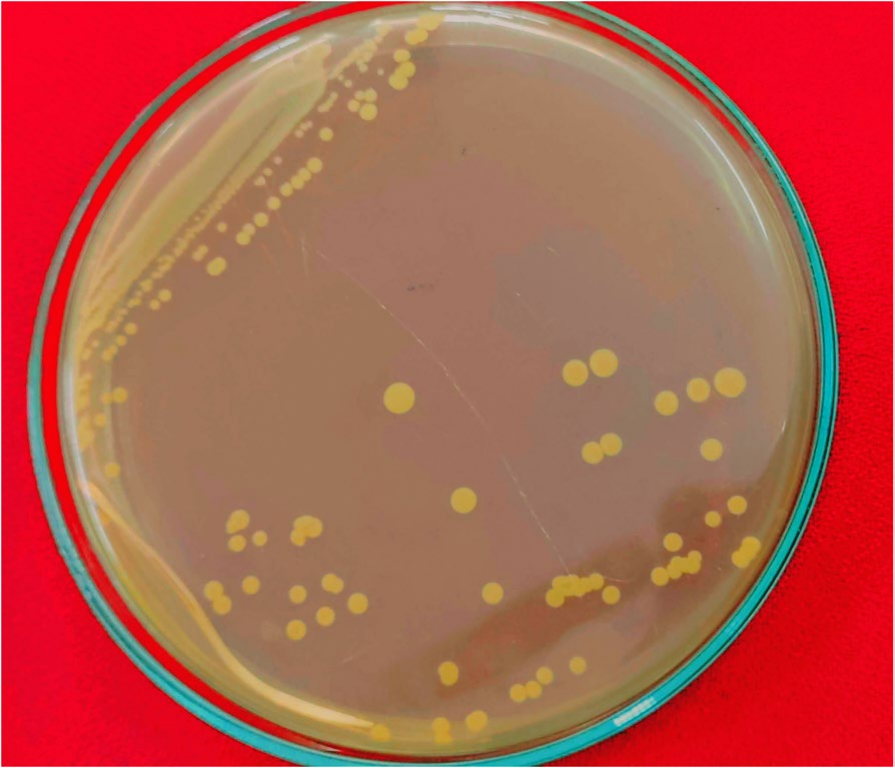
Slimy yellowish colonies of *Brachybacterium paraconglomeratum* MTCC13074 (M4) on minimal media (before Sudan staining)

**Fig. 2:**
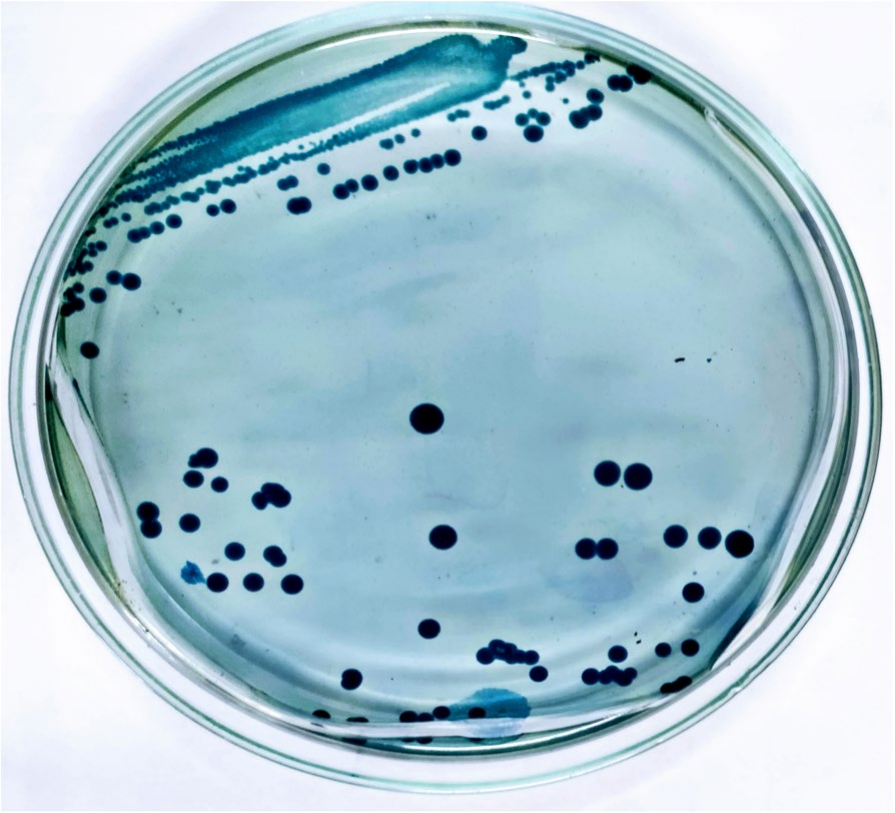
Selective staining with Sudan black B which showed positive for PHA formation with bluish black color colonies

**Fig. 3:**
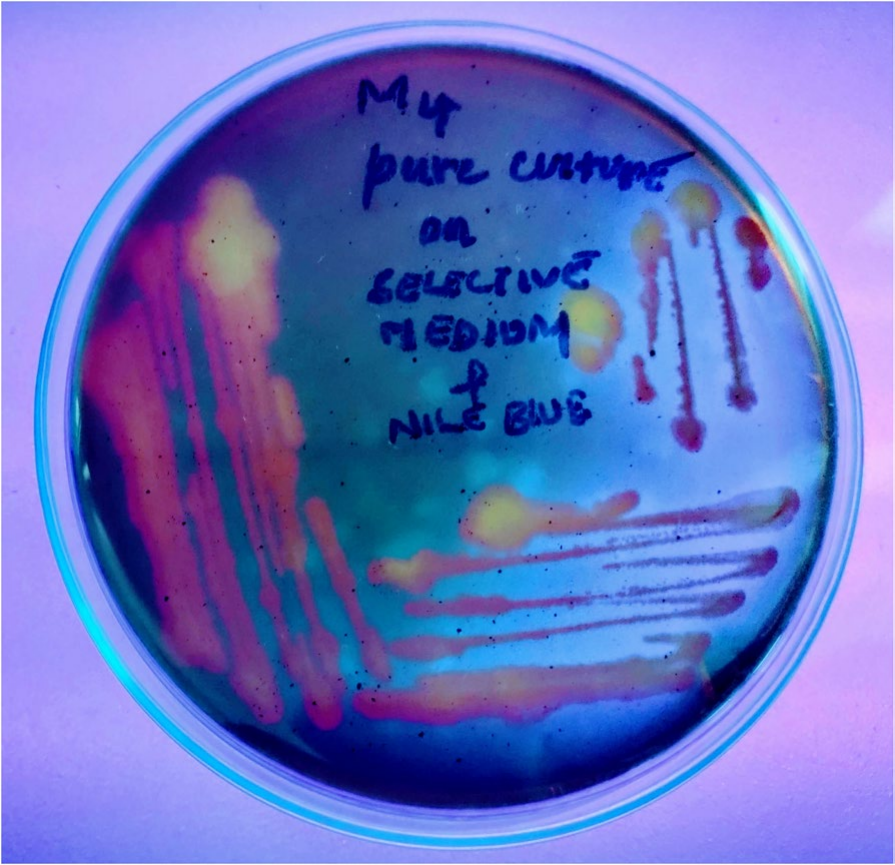
Selective screening of bacteria with PHA granules showed positive results by emitting yellow orange fluorescence with Nile blue A stain on minimal agar

The mud samples were collected from the estuary (15.858952 N, 80.511521 E) at coastal region of Bay of Bengal, Suryalanka, Bapatla, South India. Ten bacterial isolates with varied features in size shape color were identified and cultured on minimal media, only six isolates were found to be active on minimal media. On this selective media, M4 shows peculiar features like color (Fig. 1) and irregular cell shapes during differential staining. The M4 isolate was tested for PHA granules using Sudan black B stain in presumptive test. The colonies remain bluish black color (Fig. 2) after washing with 99% alcohol. The isolate M4 was cultured on Nile blue A agar plates, and after 3days of incubation at 37°C, the colonies emit orange yellowish (Fig. 3) fluorescence under UV transilluminator. The colonial features of isolate M4 are described in Table 1.

**Table 1:** Phenotypical features of M4 strain

**A**. Colonial characteristics of isolate M4	Observations
Size	Medium
Shape	Circular
Surface	Raised surface
Margin	Entire (Smooth)
Pigmentation	Pale yellow
Texture	Mucoid
Opacity	Slightly transparent
**B**. Gram staining	Gram positive, cocci

### Biochemical activities of the isolate M4

The biochemical activities of the isolate M4 are shown in Table 2. The oxidase, urease, vogues proskauer, nitrate reduction, and motility tests all came back negative, but acid production was observed with 6 sugars during fermentation. At 37°C, maximum growth was observed. M4 has a high ability to hydrolyze substrates (starch, gelatin, casein). M4 is slightly halophilic that allows it to grow up to 3.5% of NaCl concentration. The same strain exhibits γ-hemolysis. The results were compared to the data in Bergey’s Manual of Determinative Bacteriology and the isolate was identified as a facultative anaerobe, non-motile, and coccoid in shape. The isolate M4 was then subjected to 16S rRNA molecular sequencing for further characterization.

**Table 2:** Biochemical activities of M4

Name of the activity	M4
Methyl red	+
Catalase	+
Oxidase	−
Vogues proskauer	−
Urease	−
Hemolysis	γ
Indole	−
Nitrate reduction	−
Starch hydrolysis	+
Gelatin hydrolysis	+
Casein hydrolysis	+
Motility	−
Carbohydrate fermentation tests	
1. Glucose	+
2. Sucrose	+
3. Maltose	+
4. Fructose	+
5. Lactose	+
6. Raffinose	+
Growth at pH 4.2	−
Growth at pH 9.2	−
Growth at 35 to 38°C	+
Growth in NaCl (up to 3.5%)	+

+ is positive, − is negative, γ is γ-hemolysis

### Fatty acid methyl ester analysis

FAME analysis distinguishes bacterial species more quickly and easily than biochemical characterization. The analysis was performed using GC; FAMEs (fatty acid methyl esters) were formed by methanolysis in acidic conditions. The principal cellular fatty acids were C14:0, C15:0, C16:0, C18:1cis-9, C18:0, iso-C15: 0, iso-C14: 0, anteiso-C17: 0, and C18:1ω-7, and 2 unidentified phospholipids are detected and listed in Table 3. In this study, basing on area %, octane 9 enoic acid was identified as major lipid and (11E)-11- octadecanoic acid was detected as less % of lipid in the M4 strain

**Table 3:** Fatty acid methyl ester profile of *B. paraconglomeratum* MTCC13074 (M4)

S. No.	Retention time (min)	Area %	Fatty acid	Name of the fatty acid
1	17.546	4.05	C14:0	Tetradecanoic acid, 12- methyl-, methyl ester
2	18.137	12.48	C15:0	Pentadecanoic acid
3	18.515	7.96	C16:0	n-Hexadecanoic acid
4	20.328	21.06	C18:1cis-9	Octadec-9-enoic acid
5	20.454	6.25	C18:0	Octadecanoic acid
6	18.275	10.18	iso-C_15 : 0_	Isopentadecanoic acid
7	15.12	5.04	iso-C_14 : 0_	Isomyristic acid
8	20.120	6.30	anteiso-C_17 : 0_	14-Methylhexadecanoic acid, anteiso
9	20.590	2.08	C18:1ω-7	(11*E*)-11-octadecenoic acid

### 16SrRNA sequence of M4 strain

**Fig. 4:**
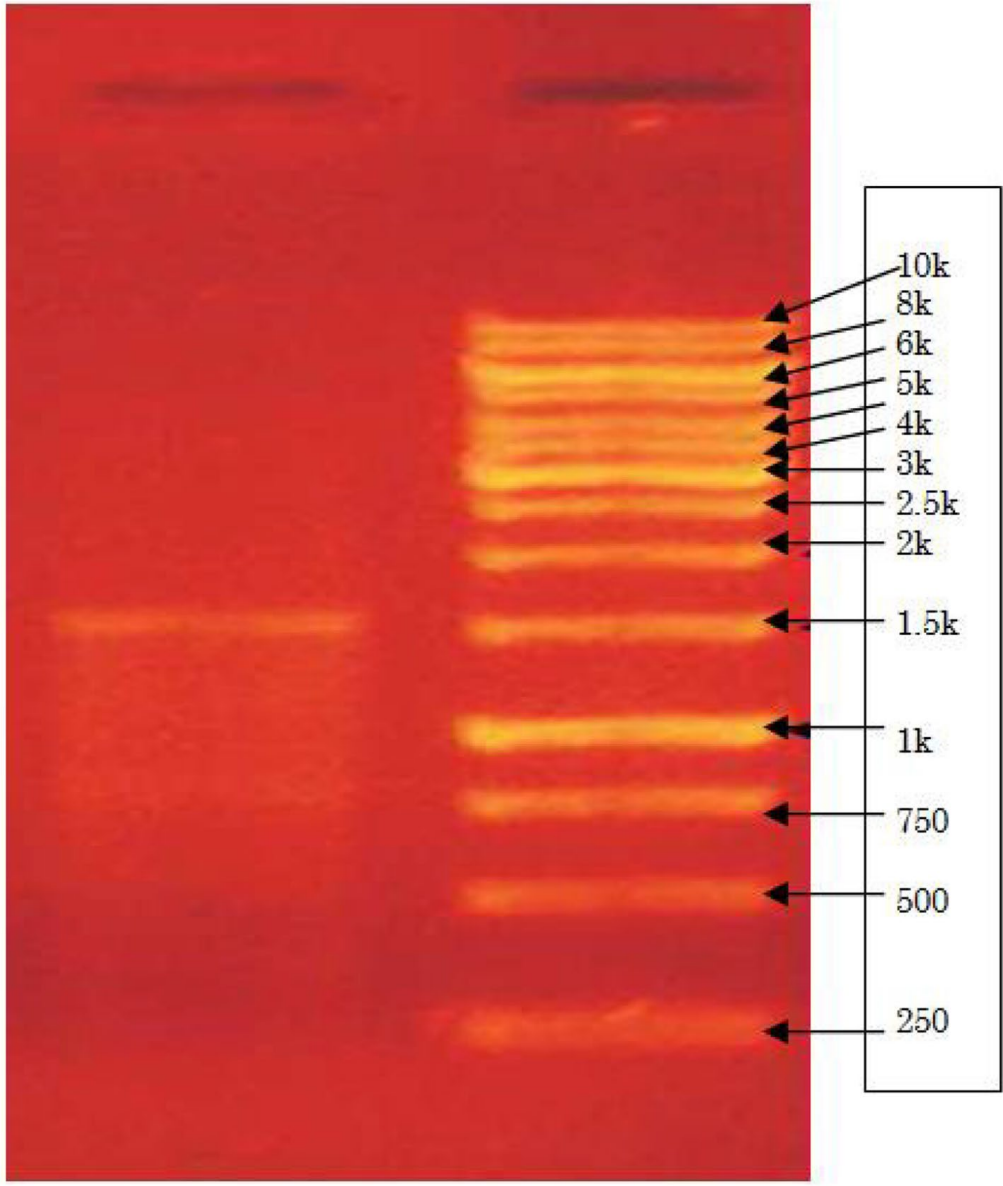
Agarose gel electrophoresis of PCR amplified product (standard DNA ladder of 10kb)

**Fig. 5:**
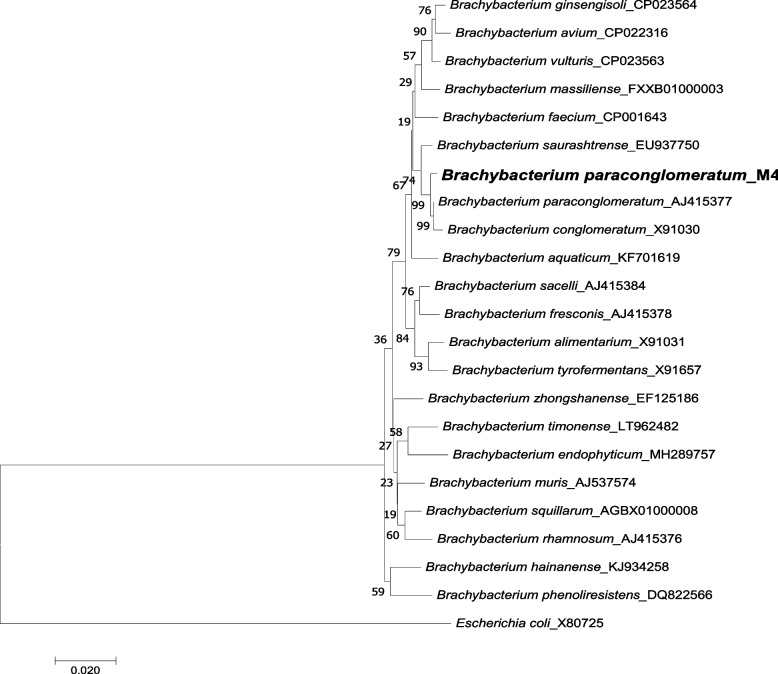
Phylogenetic tree design for bacterial culture M4 (*Brachybacterium paraconglomeratum*) using the neighbor joining method in MEGA7 software using 16S rRNA gene sequence.

DNA was extracted from M4, and 1% agarose gels (Fig. 4) was used to check the homogeneity. 16S rRNA analysis is an unambiguous identification to classify bacteria at the species level. Using universal primers, the 16S rRNA sequence was amplified. On agarose gels, a single separated PCR product of 1500 bp was detected and the PCR product was refined to remove impurities. The sequence obtained was BLAST searched to retrieve the first ten hits and tabulated (Table 4). The % identity among the top ten closely related species after the BLAST ranged from 99.72 to 97.67%. Using aligner software, the generated consensus sequence was created using forward and reverse sequence data. A phylogenetic tree (Fig. 5) was designed for bacterial culture M4 (*Brachybacterium paraconglomeratum*) based on 16S rRNA gene sequences using neighbor joining method in MEGA7 software. Isolate M4 was deposited in CSIR-IMTech and accessed as *Brachybacterium paraconglomeratum* MTCC 13074 (M4). The 16S ribosomal sequence was deposited in GenBank and NCBI, and the accession number assigned is MW899045.

**Table:** 

**>MTCC13074** ***Brachybacterium paraconglomeratum*** **strain M4 16S ribosomal RNA** sequenceCACATGCAAGTCGAACGATGACGGTGGTGCTTGCACCGCCTGATTAGTGGCGAACGGGTGAGTAACACGTGAGTAACCTGCCCCCCACTTCGGGATAACCTCGGGAAATCGTGGCTAATACCGGATATGAGCACTCATCGCATGGTGAGTGCTGGAAAGATTTATCGGTGGGGGATGGGCTCGCGGCCTATCAGTTTGTTGGTGAGGTGATGGCTCACCAAGACGATGACGGGTAGCCGGCCTGAGAGGGCGACCGGCCACACTGGGACTGAGACACGGCCCAGACTCCTACGGGAGGCAGCAGTGGGGAATATTGCACAATGGGCGAAAGCCTGATGCAGCGACGCCGCGTGGGGGATGACGGCCTTCGGGTTGTAAACCCCTTTCAGTAGGGAAGAAGCGAGAGTGACGGTACCTGCAGAAGAAGCGCCGGCTAACTACGTGCCAGCAGCCGCGGTAATACGTAGGGCGCAAGCGTTGTCCGGAATTATTGGGCGTAAAGAGCTTGTAGGTGGCTTGTCGCGTCTGCCGTGAAAACCCGAGGCTCAACCTCGGGCGTGCGGTGGGTACGGGCAGGCTAGAGTGTGGTAGGGGAGACTGGAACTCCTGGTGTAGCGGTGAAATGCGCAGATATCAGGAAGAACACCGATGGCGAAGGCAGGTCTCTGGGCCATTACTGACACTGAGAAGCGAAAGCATGGGTAGCGAACAGGATTAGATACCCTGGTAGTCCATGCCGTAAACGTTGGGCACTAGGTGTGGGGGACATTCCACGTTTTCCGCGCCGTAGCTAACGCATTAAGTGCCCCGCCTGGGGAGTACGGCCGCAAGGCTAAAACTCAAAGGAATTGACGGGGGCCCGCACAAGCGGCGGAGCATGCTGATTAATTCGATGCAACGCGAAGAACCTTACCAAGGCTTGACATGCACTGGACGGCTGCAGAGATGTGGCTTTCTTTGGACTGGTGCACAGGTGGTGCATGGTTGTCGTCAGCTCGTGTCGTGAGATGTTGGGTTAAGTCCCGCAACGAGCGCAACCCTCGTTCTATGTTGCCAGCACGTGATGGTGGGGACTCATAGGAGACTGCCGGGGTCAACTCGGAGGAAGGTGGGGACGACGTCAAATCATCATGCCCCTTATGTCTTGGGCTTCAAGCATGCTACAATGGTCGGTACAATGGGTTGCGAAACTGTGAGGTGGAGCGAATCCCAAAAAGCCGGCCTCAGTTCGGATTGGGGTCTGCAACTCGACCCCATGAAGTCGGAGTCGCTAGTAATCGCAGATCAGCAACGCTGCGGTGAATACGTTCCCGGGCCTTGTACACACCGCCCGTCAAGTCACGAAAGTCGGTAACACCCGAAGCCAGTGGCCCATCCTCGTGAGGGAGCTGTCGAAGGTGGGATCGGTGATTGGGACTAAGTCGTAACAGGGTTAACCCGTAA

**Table 4:** BLAST results of M4 16SrRNA sequence

Hit taxon name	Hit strain name	Accession	% Identity
*Brachybacterium paraconglomeratum*	LMG 19861(T)	AJ415377	99.72
*Brachybacterium conglomeratum*	NCIB 9859(T)	X91030	99.36
*Brachybacterium saurashtrense*	JG 06	EU937750	99.08
*Brachybacterium aquaticum*	KWS-1(T)	KF701619	98.35
*Brachybacterium faecium*	DSM 4810(T)	CP001643	98.24
*Brachybacterium sacelli*	LMG 20345(T)	AJ415384	98.03
*Brachybacterium massiliense*	mt5(T)	FXXB01000003	98.03
*Brachybacterium vulturis*	VM2412(T)	CP023563	97.74
*Brachybacterium fresconis*	LMG 20336(T)	AJ415378	97.74
*Brachybacterium ginsengisoli*	DCY80(T)	CP023564	97.67

### Estimation of crotonic acid and PHB

**Fig. 6.**
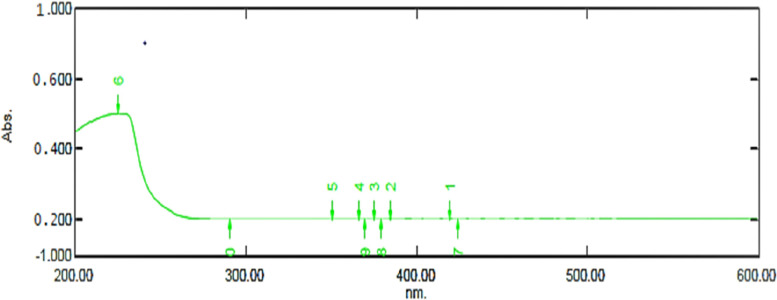
UV spectra confirmation for crotonic acid at 237nm

The PHA extracts treated with conc.H_2_SO_4_ were measured at the range of 200–600nm according to Slepecky R A method. The concentration was estimated as 2.1 *μ*g/ml in the extracts of *B. paraconglomeratum* at 237nm (Fig. 6). The total estimated biomass in 1L of culture media was 11.31g/L. Finally, the yield of PHB was estimated to be 129 mg/gdcw (w/w) represented in Table 5. The yield of PHB would be increased by optimizing the parameters of the culture media.

**Table 5:** PHB production from *B. paraconglomeratum*

Strain	Substrate	Total biomass g/L	PHB	
*Brachybacterium paraconglomeratum*	Dextrose at 1%	11.31	mg/g of dcw	g/l
			129	1.465

### GC-MS detection of biopolymers

**Fig. 7:**
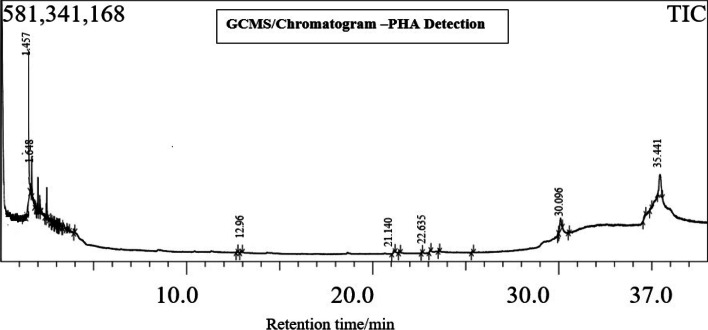
Chromatogram representing the GC confirmation for the derivatives of PHA in the extracts of *B. paraconglomeratum*

GC–MS analysis was used to confirm the derivatives of PHA/PHB. The outcomes were compared to those of standards. The chromatogram (Fig. 7) of the tested PHA extract revealed four major peaks with retention times of 21.140, 22.635, 30.096, and 35.441min, respectively. The derivatives like Tetradecanoic acid, 13-Docosenoic acid methyl ester, (Z)- Hexadecanoic acid methyl ester, and 9-Octadecenoic acid (Z)- 2-hydroxy-3-[(1-oxohexadecyl) oxy] propyl ester are identified by comparing with GC–MS library, and the same is depicted in the Table 6. This confirms that *B. paraconglomeratum* is one of the producers of polyhydroxybutyric acid (PHB).

**Table 6:** Derivatives of PHA confirmed in the GC-MS analysis

Peak	R. Time	Area	Area%	Height	A/H	Base m/z	Name
51	21.140	15087841	0.45	4477091	3.37	55.15	Tetradecanoic acid
53	22.635	7171994	0.21	3440255	2.08	55.05	13-Docosenoic acid, methyl ester, (Z)-
58	30.096	161531637	4.81	27304955	5.92	43.10	Hexadecanoic acid, methyl ester
62	35.441	378738659	11.28	48450771	7.82	57.10	9-Octadecenoic acid (Z)-, 2-hydroxy-3-[(1-oxohexadecyl)oxy]propyl ester
		3356296179	100.00	1130547317			

### Physical properties of biopolymer

#### Thermogravimetric analysis

**Fig. 8:**
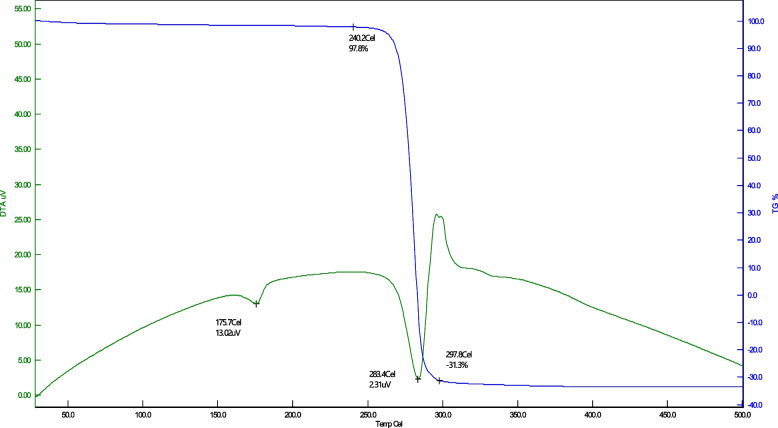
Thermogram representing the thermal nature of PHA produced by *B. paraconglomeratum*

The thermogram (Fig. 8) showed the melting temperature of extracted PHA at 175.7°C, the PHA in the extract began to lose the mass significantly from 240.2°C and decomposed completely (thermolysis) around 283.4 °C.

#### Viscosity

The viscosity was measured in 1ml of extract of different incubation hours to check the optimum time for PHA production. Data was collected and described in Table 7. Viscosity is the best physical method for detecting the presence of biopolymers in the extract. This could be considered as screening method for PHB.

**Table 7:** Viscosity of M4 extracts at different incubation times

Day 1	Day 2	Day 3	Day 4	Day 5
0.8377	0.9270	1.0369	1.1369	1.1370

Viscosity was measured at 37°C in mPa.S (Pascals)

## Discussion

Ever since petroleum-based plastics have become a major source of pollution, biodegradable plastics have gotten a lot of interest for their unique thermoplastic qualities all over the world. However, due to their greater manufacturing costs, the manufacture of these bioplastics is limited. There are already over 300 distinct bacterial species identified from various environmental conditions that produce PHA. In the current research, partial halophile *B. Paraconglomeratum* was isolated, sequenced, and screened for PHB production. As reported so far, Sudan black B and Nile blue A staining are viable colony methods that can be employed for the fast detection and isolation of PHA-producing bacteria. When stained with Sudan black B, a preliminary screening agent for lipid bodies in the bacteria, the isolates showed a black-blue coloration [19, 20] and when stained with Nile blue A, a particular dye for PHA granules, the isolates showed positive orange yellowish color [21]. Scanning electron microscopic analysis was done before and reported that *Brachybacterium* sp. are oval-shaped cells [22, 23]. Similar, biochemical tests were done and reported for *Brachybacterium* sp. [24]. The fame analysis on different *Brachybacterium* sp*.* was done and reported by Takeuchi et al. [22]. In this study, 21 to 22% of peak area for octane 9 enoic acid was identified as major lipid and with less peak area for (11E)-11-octadecanoic acid was detected and shown in the Table 3. The similar pattern of fatty acid methyl ester analysis has been specified in levan-producing *Brachybacterium* sp. [25]. *Brachybacterium saurashtrense sp. nov.*, a halotolerant root-associated bacterium with plant growth-promoting potential, was also subjected to methyl ester analysis [26]. In the study, neighbor joining (NJ) phylogenetic analysis method was done. NJ is algorithmic and statistically consistent under many models of evolution, also it will reconstruct the true tree with high probability [27]. The phylogenetic relationship between strain KWS-1T and other related members of the genus *Brachbacterium* is shown by a NJ tree based on 16S rRNA gene (1423 base) sequences and the outgroup was *Dermabacter hominis* ATCC 49369T (=DSM 7083T) (X91034) [28]. Likewise, in *Bacillus* and *Pseudomonas* spp. 16S rRNA sequencing was done and accession numbers are assigned from NCBI [29]. At 235nm, the alpha, beta-unsaturated acids, and beta-hydroxy acids converted into crotonic acid on treatment with conc.H_2_SO_4_; this is a rapid UV spectrophotometric assay reported so far [30]. Likewise, crotonic acid is estimated in the production of short side chain-polyhydroxyalkanoates from the *Ralstonia pickettii* [31]. Cell culture was cultivated as reported previously, and the cell pellet was dried to measure the dry weight of the cell (DCW) in g/L. The difference between dry weight of the cell and dry weight of extracted PHA was used to calculate the residual biomass and % of PHA produced [32]. Similar reports, such as 3-hydroxybutyric acid methyl ester and pentadecanoic acid methyl ester, were found in the chromatogram of the PHBV standard [33]. Likewise, the 3-hydroxybutyric acid methyl ester is a monomer methyl ester of 3-HB, whereas the trimer and tetramer methyl esters of 3-HV and 3-HB are pentadecanoic acid methyl ester and hexadecanoic acid methyl ester, respectively [34]. It was reported that GC detected [butanoic acid, 2-amino-4-(methylseleno); hexanoic acid, 4-methyl-, methyl ester and hexanedioic acid, monomethyl ester] as three distinct peaks of various butenoic acid derivatives from the PHB of *P. xiamenensis* [35]. In the present study, the presence of PHB in M4 extract was confirmed by GC-MS analysis, by detecting four monomer derivatives of PHB. In TGA, the thermal stability was similar to that of PHB reported by Pillai et al. [19]. The results were consistent with those obtained from the regular PHB (Tm-176.29°C) as well as other investigations. The Tm of *Bacillus* sp. NA10 is 182.34°C and the enthalpy of PHA fusion is 83.62 J/g, according to TGA findings. The observed thermal stability of the copolymer can be attributed to the sample’s higher hydroxyvalerate content, which may improve its ductility and flexibility [34]. An increase in viscosity indicates the increased production of biopolymers. However, better viscosity values appear at optimized conditions due to high yields of PHA [36].

## Conclusion

In the present study, *Brachybacterium paraconglomeratum* a novel strain was identified from the estuary near Suryalanka Bapatla, India. 16srRNA sequencing and fatty acid methyl ester analysis was used to characterize the strain. *B. paraconglomeratum*, an actinobacter, has the unprecedented feature of producing biopolymer (PHB). It is reported for the first time that a few derivatives of polyhydroxybutyric acid (PHB) were produced by this bacterium. The strain has promising features of producing bioactive compounds which were revealed by biochemical activities. This research also helps to understand the diversity of PHA-producing bacteria isolated from marine source. Future reporting would focus on the optimization, physiochemical characteristics, and biotechnological applications of PHB produced by the *B. paraconglomeratum*.

## Data Availability

Not applicable.
